# miR-346 Regulates Osteogenic Differentiation of Human Bone Marrow-Derived Mesenchymal Stem Cells by Targeting the Wnt/β-Catenin Pathway

**DOI:** 10.1371/journal.pone.0072266

**Published:** 2013-09-04

**Authors:** Qing Wang, Jie Cai, Xian-hua Cai, Lei Chen

**Affiliations:** Department of Orthopaedics Surgery, Wuhan General Hospital of Guangzhou Command, Wuhan, China; Georgia Regents University, United States of America

## Abstract

Osteogenic differentiation of human mesenchymal stem cells (hMSCs) is regulated by multiple transcription factors and signaling molecules. However, the molecular mechanisms underlying this process remain to be fully elucidated. MicroRNAs (miRNAs) act as key regulators in various biological processes by mediating mRNA degradation or translational inhibition of target genes. In this study, we report that miR-346 plays critical roles in regulating osteogenic differentiation of hBMSCs. The expression of endogenous miR-346 was increased during osteogenic differentiation of hBMSCs. Overexpression of miR-346 significantly promoted osteogenic differentiation, whereas miR-346 depletion suppressed this process. Further studies confirmed that miR-346 directly targeted the 3′-UTR of the glycogen synthase kinase-3β (GSK-3β) gene so as to suppress the expression of GSK-3β protein. Similar to miR-346 overexpression, GSK-3β depletion promoted osteogenic differentiation, whereas GSK-3β overexpression reversed the promotional effect of miR-346. We further found that miR-346 overexpression activated the Wnt/β-catenin pathway and increased the expression of several downstream genes including CyclinD1, c-Myc, TCF-1 and LEF-1. Depletion of β-catenin almost completely blocked the positive role of miR-346 on osteogenic differentiation. Taken together, our data indicate that miR-346 positively regulates hBMSC osteogenic differentiation by targeting GSK-3β and activating the Wnt/β-catenin pathway.

## Introduction

Human bone marrow-derived mesenchymal stem cells (hBMSCs) are multipotent cells that have significant clinical potential in cell-based therapeutic strategies for regeneration of various tissues. hBMSCs can differentiate into a variety of cell types including osteoblasts [1], and this process is regulated by a number of regulatory factors and complex signaling pathways, including Wnt/β-catenin pathway [2].

The canonical Wnt/β-catenin pathway is initiated by binding of Wnts (eg, Wnt3a) to the cell surface molecules LRP5/6 and Frizzled (FZD), resulting in the release of cytoplasmic β-catenin from a protein complex consisting of Axin1/2, APC, casein kinase 1 (CK1), and glycogen synthase kinase 3β (GSK3β) [3]. Upon dephosphorylation and release, β-catenin subsequently translocates into the cell nucleus. In the nucleus, β-catenin interacts with the T-cell factor/lymphoid enhancer factor-1 (TCF/LEF1) family of transcription factors and activates the expression of target genes which are necessary for cell proliferation and differentiation [4], [5].

MicroRNAs (miRNAs) are noncoding RNA molecules that negatively regulate the expression of target genes by either mRNA degradation or translational inhibition [6]. The known functions of miRNAs include a range of biological processes, including cell differentiation, proliferation, apoptosis, and tissue development [7]–[9]. In particular, miRNAs play critical roles in regulating osteogenic differentiation of mesenchymal stem cells [10]–[12]. For example, miR-138, miR-204, and miR-20a have been reported to regulate osteoblast differentiation by targeting various osteoblast genes [13]–[15]. However, more evidence for the roles of miRNAs in regulating osteogenic differentiation is needed.

In view of the importance of miRNAs in the regulation of osteoblast differentiation, we wondered whether the Wnt/β-catenin pathway might be regulated by miRNAs during osteoblast differentiation of hBMSCs. We performed microarray analysis and identified miR-346 as a noncoding RNA that directly binds to the 3′-untranslated region (UTR) of GSK-3β mRNA. The aim of the present study was to validate the regulatory relationship between miR-346 and GSK-3β in hBMSCs and to investigate the role of this mechanism in osteogenic differentiation. Our findings suggest that miR-346 promotes osteogenic differentiation by repressing GSK-3β and activating the Wnt/β-catenin pathway.

## Materials and Methods

### Isolation and culture of hBMSCs

hBMSCs were isolated from human bone marrow as previously described [16]. In brief, human bone marrow samples were aspirated from 3 healthy donors. The study was approved by the Ethics Committee of Wuhan General Hospital of Guangzhou Command, People's Liberation Army, and written informed consent was obtained from each donor. Mononuclear cells were isolated on a Ficoll density gradient, and cultured in Minimum Essential Medium Alpha Medium (α-MEM) supplemented with 17% (vol/vol) FBS, 2 mM L-Glutamine, 100 U/mL penicillin and 100 μg/mL streptomycin at 37°C with 5% humidified CO_2_. After 24 h, the non-adherent cells were removed, and the adherent cells were further cultured in complete medium until the cells were approximately 80% confluent. hBMSCs from passage 3 to passage 5 were utilized for this study.

### Osteogenic differentiation

A total of 2×10^5^ hBMSCs were plated into each well of a 6-well plate and cultured. At 80% confluence, the medium was replaced with complete medium supplemented with 10 nM dexamethasone, 0.2 mM L-ascorbic acid, and 10 mM β-glycerophosphate to induce osteogenicdifferentiation. The cells were cultured in differentiation medium for 15 days with a medium change every 3 days. After hBMSCs were fixed in 4% paraformaldehyde for 10 min, the osteoblast phenotype was evaluated by determining ALP activity. ALP activity and ALP staining were performed using an alkaline phosphatase detection kit (Jiancheng Bioengineering, Nanjing, China) and an ALP staining kit (Blood institute, Chinese Academy of Medical Sciences) according to the manufacturers' suggested protocols. Alizarin Red staining was performed to detect matrix mineralization with 2% Alizarin Red S (ARS; Sigma, St. Louis, MO, USA), pH 4.2, for 10 min at room temperature. Each experiment was repeated in triplicate.

### RNA isolation and quantitative real-time PCR (qRT-PCR)

Total RNA was extracted from cells with Trizol reagent (Invitrogen, Carlsbad, CA, USA). cDNA was synthesized using the PrimeScript RT reagent Kit (TaKaRa, Dalian, China). TaqMan miRNA assays were used to evaluate the expression of miR-346, with U6 as an internal control. Human Runx2, ALP, OPN and GSK-3β transcripts were quantified by qRT-PCR using the SYBR Premix Ex Taq II kit (TaKaRa) and the Applied Biosystems ABI Prism 7500 HT sequence detection system, with β-actin as an internal control. The primers for Runx2 were 5′-TCTTCACAAATCCTCCCC-3′ (forward) and 5′-TGGATTAAAAGGACTTGG-3′ (reverse). The primers for ALP were 5′-ACGTGGCTAAGAATGTCATC-3′ (forward) and 5′-CTGGTAGG

CGATGTCCTTA-3′ (reverse). The primers for OPN were 5′-ACTCGAACGACTCTGATGATGT

-3′ (forward) and 5′-GTCAGGTCTGCGAAACTTCTTA-3′ (reverse). The primers for GSK-3β were 5′-GCTTTGAAAGTAATCCCTGGGGTTTGG-3′ (forward) and 5′-TGCAGAGGTGCAAA

ACGGAGCA-3′ (reverse). Expression of mRNA or miRNA was evaluated by the 2^−△△Ct^ method and normalized to the expression of β-actin or U6 respectively. All reactions were run in triplicate.

### Lentivirus infection and oligonucleotide transfection

The miR-346, anti-miR-346 and GSK-3β siRNA were purchased from Ambion Qiagen (Valencia, CA, USA). The constructs containing the pre-miR-346 or GSK-3β siRNA sequence were cloned into the lentivirus-based expression plasmid pLenti-6.3 (Invitrogen). Lentivirus was packaged following the manufacturer's protocol. Viruses were harvested 48 hours after transfection and viral titers were 2×10^9^ TU/mL. hBMSCs (1×10^5^) were infected with 1×10^7^ recombinant lentivirus-transducing units plus 8 mg/mL Polybrene (Sigma). Empty lentiviral vector was used as the negative control. To knock down miR-346 in hBMSCs, cells were transfected with anti-miR-346 (at a final concentration of 100 nM) or anti-NC using Lipofectamine 2000 (Invitrogen) according to the manufacturer's protocol. Cells were collected 48 h after transfection.

### Plasmid construction

The full-length open reading frame of GSK-3β was cloned into pcDNA3.1 (+) to generate GSK-3β expression vectors. The primers for GSK-3β were 5′-CGTGAATTCTCGCGAAGAGAG

TGATCATGTC-3′ (forward) and 5′-CTCTCTAGAAAGCAGCATTATTGGTCTGTCC-3′ (reverse). The wild-type GSK-3β 3′UTR (WT) was cloned into the pGL3-Control Vector (Promega, Madison, WI, USA). Site-directed mutagenesis of the miR-346 seed sequence in the GSK-3β 3′-UTR (Mut) was performed using the QuikChange™ Site-Directed Mutagenesis Kit (Stratagene, La Jolla, CA, USA). The primers for GSK-3β 3′-UTR were 5′-CCACTCGAGGTGG

CGTTGATAGACCATTTTC-3′ (forward) and 5′-CATGCGGCCGCACACACACACACACGCA

CACAT-3′ (reverse).

### Luciferase reporter assays

For GSK-3β 3′-UTR luciferase reporter activity assays, stable miR-346-overexpressing HEK293T and hBMSCs were cultured in 24-well plates, and transfected with 100 ng luciferase reporter plasmid and 5 ng pRL-TK vector expressing Renilla luciferase (Promega) using Lipofectamine 2000 (Invitrogen). For analysis of TCF/LEF transcriptional activity, miR-346-overexpressing hBMSCs were transfected with 200 ng TOPflash or FOPflash (Millipore, Billerica, MA, USA) and 5 ng pRL-TK (Promega) using Lipofectamine 2000 (Invitrogen). After 48 h, cells were harvested and lysed, and luciferase activity was measured using the Dual-Luciferase Reporter Assay System (Promega). Renilla-luciferase was used for normalization. The experiments were performed independently in triplicate.

### Western blotting

Cells were lysed with radioimmunoprecipitation assay lysis buffer and total protein content was collected by centrifugation. Protein concentration was determined using the BCA protein assay kit (Pierce, Rockford, IL, USA) according to the manufacturer's protocol. A 20 µg aliquot of lysate proteins were fractionated by sodium dodecyl sulfate polyacrylamide gel electrophoresis and subsequently transferred to a nitrocellulose membrane, which was blocked with 5% non-fat dry milk for 2 h. Membranes were then incubated overnight at 4°C with primary antibodies against human active-β-catenin (Millipore, Billerica, MA, USA), GSK-3β, β-catenin, c-Myc, TCF-1, LEF-1, CyclinD1, and β-actin (Santa Cruz Biotechnology, Inc., Santa Cruz, CA, USA). Protein bands were detected by incubation with horseradish peroxidase-conjugated antibodies and visualized with an enhanced chemiluminescence reagent (Amersham Biosciences, Piscataway, NJ, USA).

### Immunofluorescence localization

Cells were fixed in 4% paraformaldehyde for 10 min, penetrated with 0.1% Triton X-100 and blocked with 5% bovine serum albumin for 1 h. Then, the cells were incubated with primary antibodies against β-catenin at 4°C overnight, followed by incubation with anti-mouse Rhodamine-linked IgG (Santa Cruz). Once labeled, the cells were detected under an Olympus IX71 fluorescence microscope. Nuclei were counterstained with 4′,6-diamidino-2-phenylindole (DAPI, 0.1 µg/mL).

### Statistical analysis

Data are expressed as mean ± SD from at least three independent experiments. Comparisons were performed using a two-tailed t test or one-way ANOVA for experiments with more than two subgroups. *p*<0.05 was considered statistically significant.

## Results

### Expression of miR-346 during osteogenic differentiation of hBMSCs

Previous studies have shown that activation of the canonical Wnt/β-catenin signaling pathway by inhibition of glycogen synthase kinase 3 (GSK-3) could enhance osteoblastic differentiation [17], [18]. To search for regulators of the Wnt/β-catenin pathway, we screened miRNAs during osteogenic differentiation of hBMSCs using human miRNA microarrays (Agilent Technologies, Santa Clara, CA, USA). In combination with bioinformatics analyses using Targetscan and miRanda, we identified miR-346 as a potential regulator binding to the 3′-UTR of GSK-3β mRNA.

**Figure 1:**
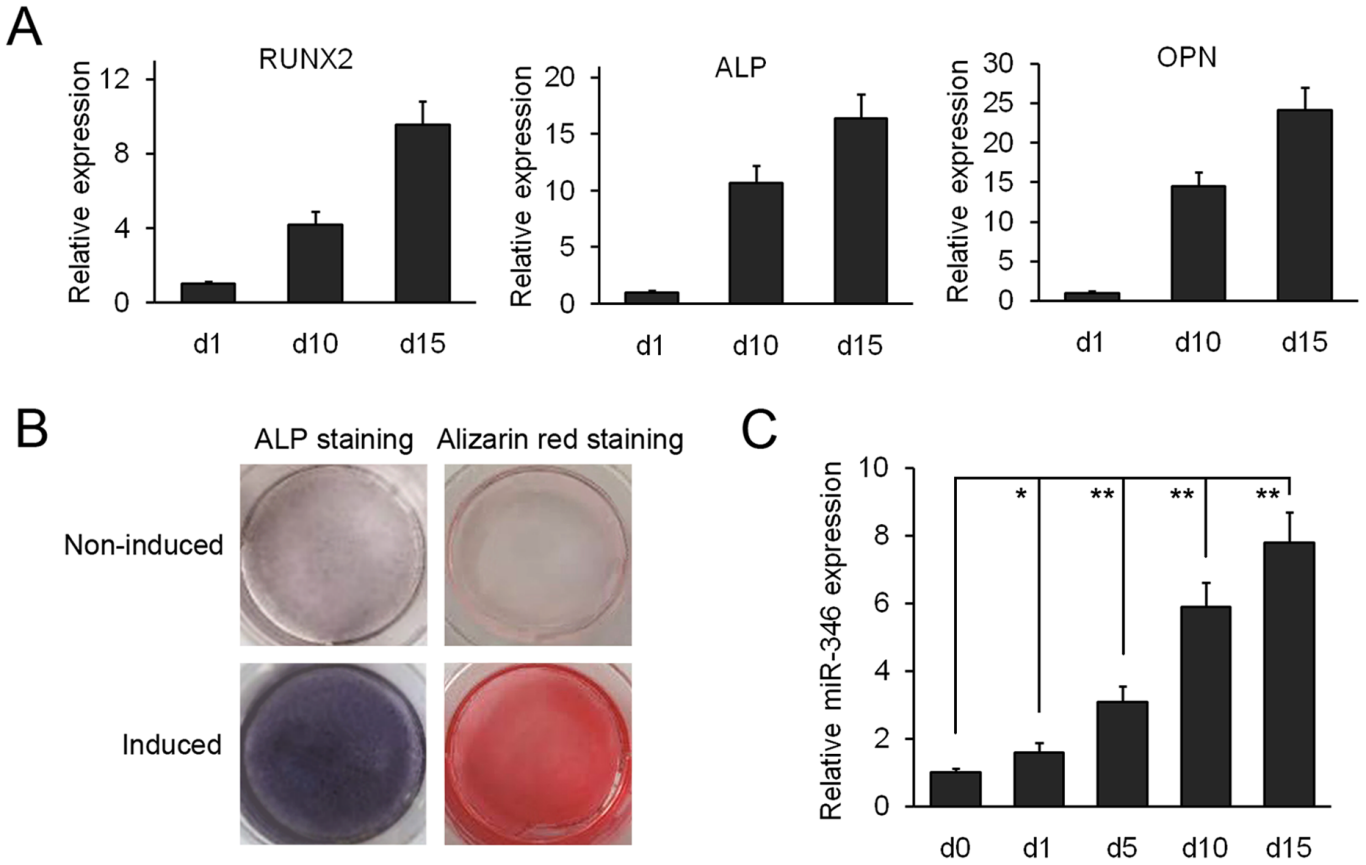
Expression levels of miR-346 during osteogenic differentiation of hBMSCs. (A) qRT-PCR analysis of the osteoblastic marker genes *RUNX2*, *ALP*, and *OPN*. *β-actin* was used as an internal control. (B) ALP staining and Alizarin Red staining were performed at day 10 and day 15, respectively. (C) The expression of miR-346 during osteogenic differentiation of hBMSCs was measured by qRT-PCR. U6 was used as an internal control. All data are presented as mean ± SD from 3 independent experiments with cells from 3 different donors. *p<0.05, **p<0.01.

To examine whether miR-346 is involved in osteogenic conversion, hBMSCs were cultured in osteogenic differentiation medium. The expression of osteoblastic marker genes including *RUNX2*, *ALP*, and *OPN*, was significantly increased after induction of differentiation (Figure 1A). Correspondingly, both ALP and Alizarin Red staining experiments confirmed the osteoblast phenotype (Figure 1B). Subsequently, the expression of miR-346 was examined at different time points during osteoblast differentiation. The results showed that endogenous miR-346 expression increased gradually during osteogenic induction differentiation of hBMSCs (Figure 1C), suggesting that miR-346 might be involved in regulating their osteogenic differentiation.

### miR-346 promotes osteogenic differentiation of hBMSCs

**Figure 2:**
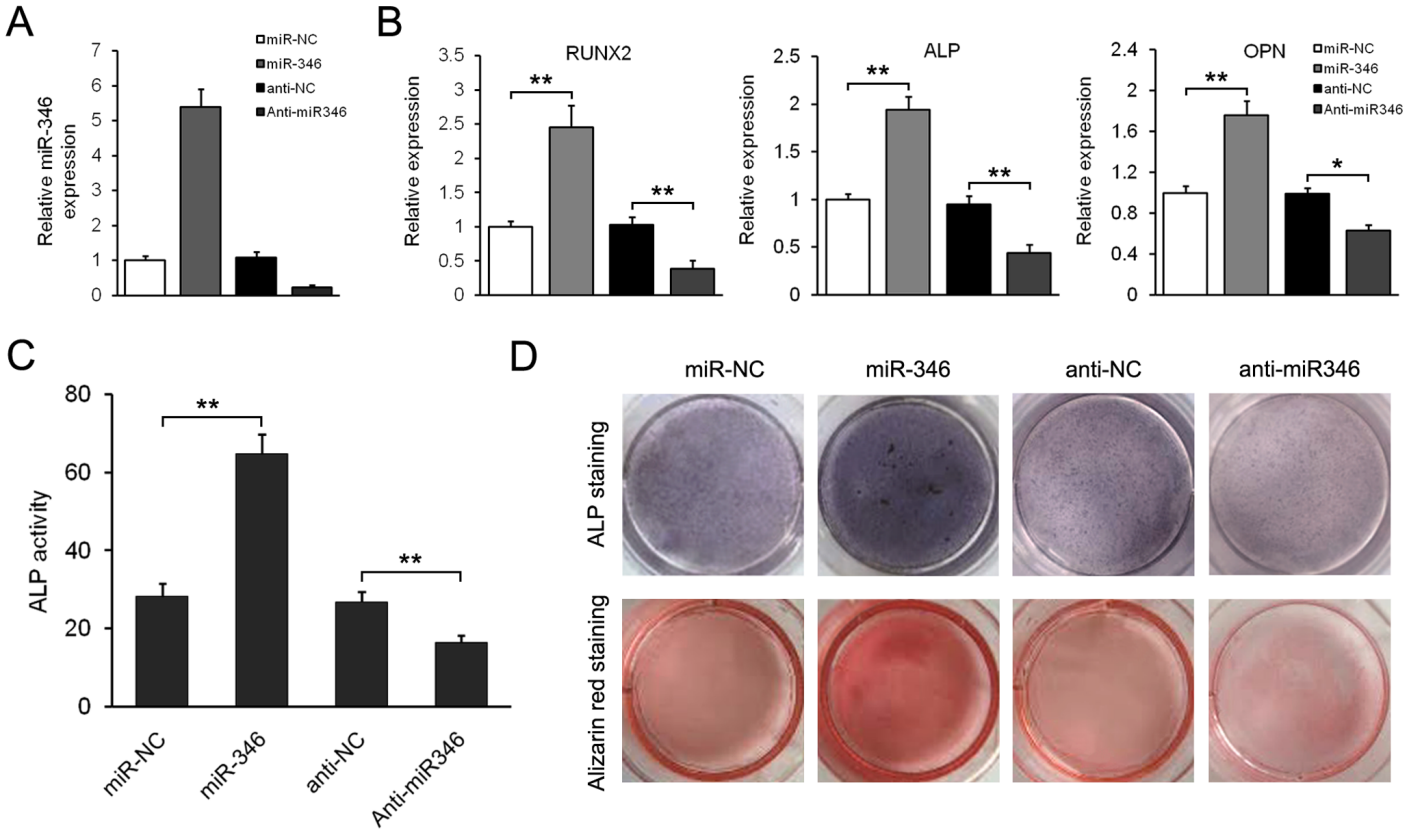
miR-346 promotes osteogenic differentiation of hBMSCs. (A) qRT-PCR analysis of miR-346 in hBMSCs infected by miR-346-lentivirus or transfected with anti-miR346. The data were normalized to U6. (B) qRT-PCR analysis of the osteoblastic marker genes *RUNX2*, *ALP*, and *OPN* at day 15 of osteoblast differentiation. The data were normalized to *β-actin*. (C) ALP activity was measured at day 10. (D) ALP staining and Alizarin Red staining were performed at day 10 and day 15, respectively. All data are expressed as mean ± SD from 3 experiments with cells from 3 different donors. *p<0.05, **p<0.01.

To investigate the role of miR-346 in osteogenic differentiation, we constructed a lentivirus vector harboring pre-miR-346 and established stably miR-346-overexpressing hBMSCs. Increased expression of miR-346 in hBMSCs was verified by qRT-PCR (Figure 2A). We found that overexpression of miR-346 significantly increased osteoblastic differentiation, which was indicated by higher expression of osteoblastic marker genes *RUNX2*, *ALP*, and *OPN*, increased ALP activity and matrix mineralization level in miR-346-overexpressing hBMSCs compared to negative control (Figure 2B–2D). In contrast, expression of osteoblast marker genes, ALP activity and matrix mineralization level were all reduced in hBMSCs in which miR-346 was depleted using antisense oligonucleotides. These results suggest that gene transfer of miR-346 obviously promotes osteogenic differentiation of hBMSCs, while loss of endogenous miR-346 is capable of inhibiting progression of hBMSCs to differentiated osteoblastic phenotypes.

### miR-346 downregulates GSK-3β through interaction with its 3′-untranslated region

**Figure 3:**
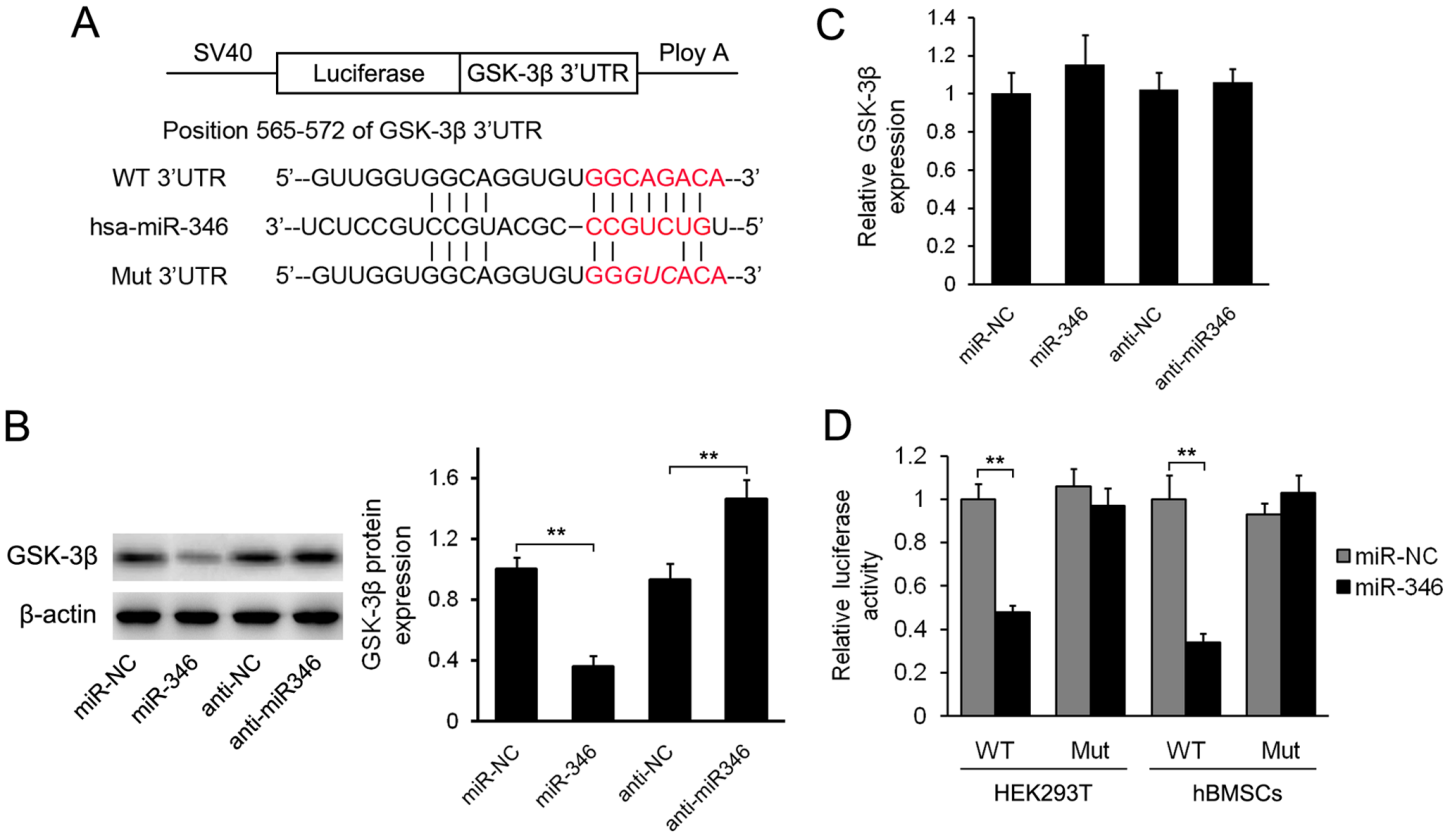
miR-346 downregulates GSK-3β by interacting with its 3′-UTR. (A) Putative miR-346 binding sequence in the GSK-3β 3′-UTR. Fragments of GSK-3β 3′-UTR containing wild-type (WT) or mutated (Mut) miR-346 binding sites were cloned into pGL3-control vector to obtain GSK-3β 3′-UTR luciferase reporter plasmids. (B) Western blotting analysis of GSK-3β expression in hBMSCs transfected with miR-346-lentivirus or anti-miR346 after 48 h. (C) qRT-PCR analysis of *GSK-3β* expression in hBMSCs infected with miR-346-lentivirus or transfected with anti-miR346 after 48 h. The data were normalized to *β-actin*. (D) The WT or Mut reporter plasmids were co-transfected into hBMSCs which were infected by miR-NC-lentivirus or miR-346-lentivirus. Relative repression of firefly luciferase expression was standardised to a transfection control. Data shown are mean ± SD from 3 experiments with cells from 3 different donors. **p<0.01.

Analysis by different computational methods (TargetScan and miRanda) indicated that GSK-3β is a candidate target of miR-346 (Figure 3A). Thus, we wondered whether miR-346 could directly down-regulate GSK-3β expression. Western blotting analysis showed that overexpression of miR-346 substantially decreased the expression of GSK-3β, while anti-miR-346 transfection increased GSK-3β protein levels (Figure 3B). Much to our surprise, qRT-PCR showed that overexpression or inhibition of miR-346 had no effect on GSK-3β mRNA level (Figure 3C), suggesting that miR-346 specifically regulates GSK-3β expression at the posttranscriptional level. To demonstrate that miR-346 binds to the 3′-UTR of GSK-3β, we next conducted luciferase assays using the constructs shown in Figure 3A. In this analysis, we observed a significant reduction of luciferase activity in a vector containing the target site of GSK-3β, whereas cells with a mutant GSK-3β 3′-UTR displayed much higher luciferase activities (Figure 3D). Taken together, these results suggest that GSK-3β is a direct target of miR-346.

### miR-346 represses GSK-3β expression to promote osteogenic differentiation

**Figure 4:**
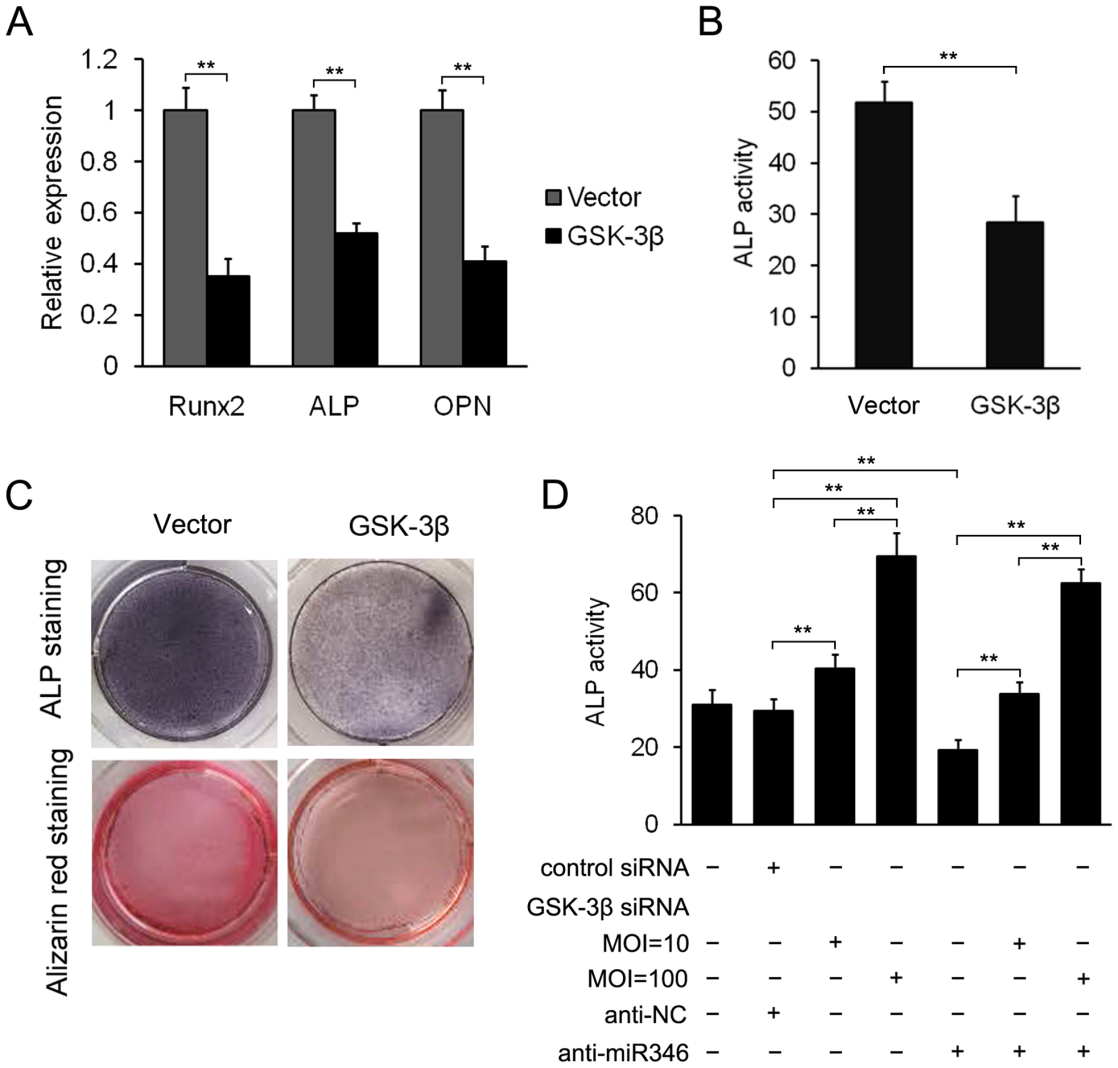
The positive regulatory effect of miR-346 on osteogenic differentiation is mediated by repression of GSK-3β. (A) GSK-3β overexpression inhibits the expression of osteoblastic marker genes in miR-346-overexpressing cells. (B) GSK-3β overexpression reduced ALP activity in miR-346-overexpressing cells. (C) ALP staining and Alizarin Red staining showed that GSK-3β overexpression suppresses osteogenic differentiation. (D) GSK-3β is required for the inhibitory effect of anti-miR-346 on ALP activity. All data represent mean ± SD of 3 independent experiments with cells from 3 different donors. **p<0.01.

To elucidate whether miR-346-induced osteogenic differentiation is mediated by repression of GSK-3β, we performed gain-of-function and loss-of-function analyses. First we examined the effect of GSK-3β overexpression on miR-346-induced osteogenic differentiation of hBMSCs. qRT-PCR analyses showed that the expression of osteoblastic marker genes, including *RUNX2*, *ALP*, and *OPN*, was suppressed by overexpression of GSK-3β in the miR-346-overexpressing cells (Figure 4A). Accordingly, GSK-3β overexpression reduced ALP activity and matrix mineralization evaluated by ALP and Alizarin Red staining (Figure 4B and C). Subsequently, we found that inhibition of GSK-3β by GSK-3β siRNA significantly enhanced osteoblastic differentiation, indicated by increased ALP activity, and that anti-miR-346 significantly downregulated ALP activity in hBMSCs (Figure 4D). In addition, cotransfection of GSK-3β siRNA with anti-miR-346 almost completely blocked the inhibitory effect of anti-miR-346 on ALP activity (Figure 4D), and increased amounts of GSK-3β siRNA led to higher ALP activity, suggesting that the effect of miR-346 on ALP activity is GSK-3β-dependent. Taken together, these results suggest that GSK-3β is a functional target of miR-346.

### miR-346 activates the Wnt/β-catenin pathway

**Figure 5:**
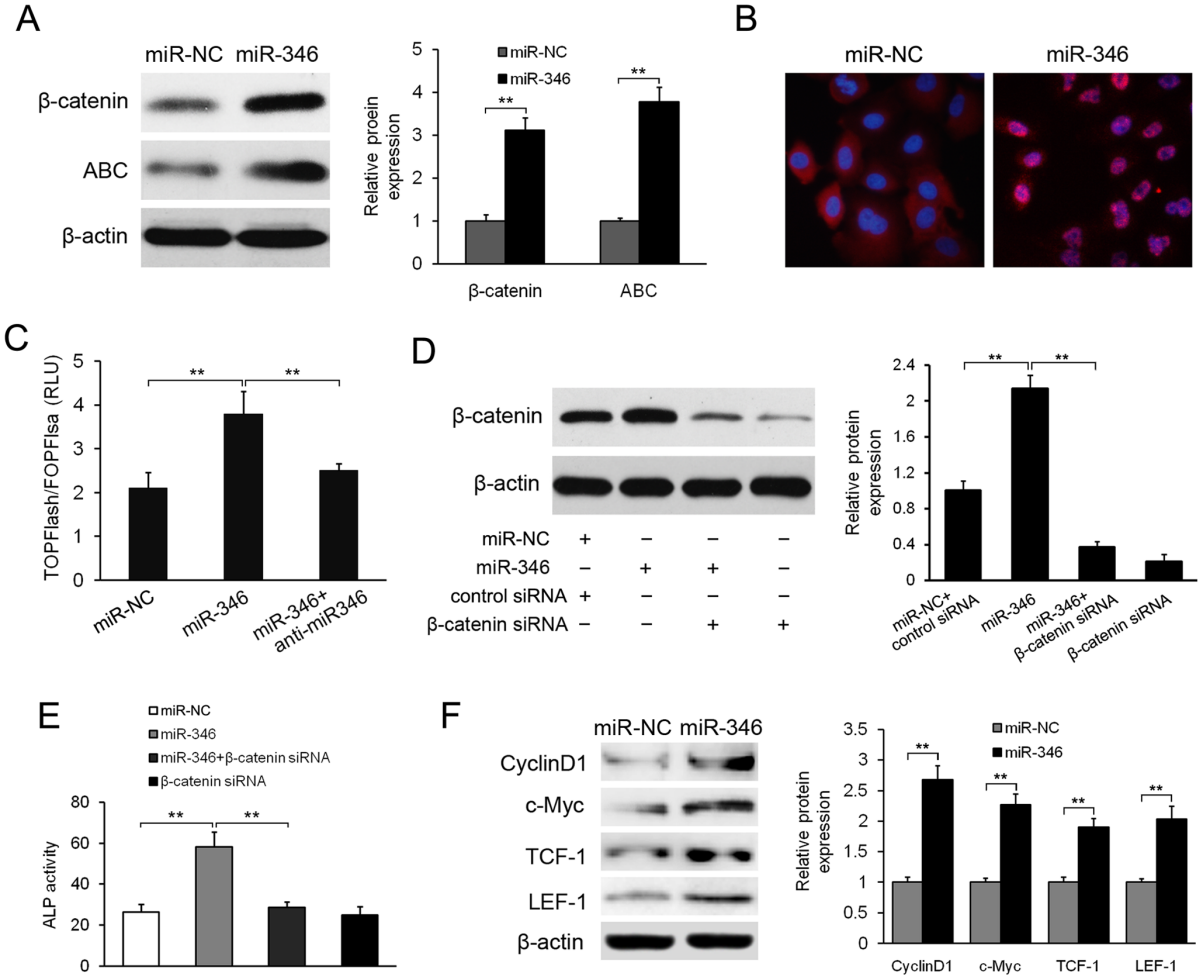
miR-346 promotes activation of the Wnt/β-catenin pathway. (A) miR-346 overexpression enhanced the expression of both total β-catenin and activated β-catenin (ABC) in hBMSCs. (B) miR-346-overexpressed cells showed more β-catenin accumulation in the nuclei compared with the control cells. (C) Luciferase activity of TOPFlash/FOPFlash in miR-346-overexpressing cells. (D) Western blotting analyses of β-catenin expression in hBMSCs infected with miR-346 or β-catenin siRNA. (E) β-catenin deletion almost completely blocked the positive effect of miR-346 on ALP activity. (F) Western blotting analyses of Wnt/β-catenin downstream molecules in miR-346-overexpressing hBMSCs. All data represent mean ± SD of 3 independent experiments with cells from 3 different donors. **p<0.01.

Given that GSK-3β mediates β-catenin degradation via phosphorylation of its serine (Ser33/37/45) and threonine (Thr41) residues, we examined the expression of total β-catenin and activated β-catenin (ABC) in hBMSCs with miR-346 overexpression. As shown in Figure 5A, a significant increase in total β-catenin and ABC expression was found. Consistently, immunofluorescence staining showed increased β-catenin nuclear accumulation in miR-346-overexpressing cells compared to negative controls. Next we sought to determine whether miR-346 could regulate the Wnt/β-catenin pathway (Figure 5B). For this purpose, miR-346-overexressing cells were transiently transfected with the Wnt signaling reporter TOPFlash or the negative control FOPFlash. We found that TCF/LEF transcriptional activity was significantly increased in miR-346-overexpressing cells, whereas this effect could be inhibited by anti-miR-346 (Figure 5C), indicating that Wnt/β-catenin signaling is altered by miR-346. To further investigate the functional role of the increased β-catenin induced by miR-346 overexpression, we performed loss-of-function analyses by stable knockdown of β-catenin in miR-346-overexpressing cells (Figure 5D). The results showed that miR-346-overexpressing cells had higher ALP activity compared to control cells, and that β-catenin knockdown in these cells almost completely blocked the positive role of miR-346 on the ALP activity (Figure 5E). We also found that miR-346 overexpression significantly increased the expression of Wnt/β-catenin downstream signaling molecules, such as c-Myc, CyclinD1, TCF-1, and LEF-1 (Figure 5F). Taken together, these results indicate that miR-346 promotes osteogenic differentiation of hBMSCs by activating Wnt/β-catenin signaling.

## Discussion

In the present study, we identified miR-346 as a positive regulator of the osteogenic differentiation of hBMSCs. We found that miR-346 was highly expressed during the course of osteogenic differentiation. Overexpression of miR-346 in hBMSCs enhanced osteogenic differentiation, whereas inhibition of miR-346 suppressed their osteogenic potential.

miRNAs, as novel regulators of target gene expression, play important roles in regulation of stem cell differentiation into osteoblasts by controlling the levels of critical factors [19], [20]. For instance, miR-138, which is down-regulated during osteoblast differentiation of hBMSCs, functions as a negative regulator of osteogenic differentiation by targeting FAK and suppressing the FAK-ERK1/2 signaling pathway [13]. miR-204 inhibits osteoblast differentiation of BMSCs, while adipocyte differentiation is promoted when miR-204 is overexpressed in these cells [14]. miR-20a promotes the osteogenesis of hMSCs in a co-regulatory pattern by targeting PPARγ, Bambi and Crim1, the negative regulators of BMP signaling [15]. Here, we report that GSK-3β is a direct target of miR-346 in hBMSCs. Our data indicate that miR-346 overexpression significantly down-regulates GSK-3β by directly targeting the 3′UTR of GSK-3β mRNA confirmed using luciferase-reporter-gene assays, and this effect was largely eliminated when the sites in GSK-3β 3′UTR targeted by miR-346 were mutated. Moreover, miR-346 overexpression in hBMSCs resulted in upregulation of GSK-3β protein level without changing its mRNA expression, confirming that miR-346 regulates GSK-3β expression at a post-transcription level.

GSK-3β, an isoform of GSK-3, is implicated in various biological processes including cell growth, differentiation and apoptosis [17], [21]. Mounting evidence indicates that GSK-3β inhibition promotes bone formation in vivo [22], [23]. In recent years, GSK-3β has been reported to play important roles in regulating osteoblast differentiation. Gambardella *et al*. [17] have shown that inhibition of GSK-3β promotes osteogenic differentiation of mesenchymal progenitors but not adipogenic differentiation. Jang *et al*. [24] found that GSK-3β inactivation upon receptor activator of NF-κB ligand (RANKL) stimulation is crucial for osteoclast differentiation. In this study, we demonstrate that GSK-3β is a functional target of miR-346. Our data show that osteogenic differentiation of hBMSCs is suppressed by overexpression of GSK-3β inmiR-346-overexpressing cells, while GSK-3β siRNA almost completely blockes the inhibitory role of anti-miR-346 on osteogenic differentiation, suggesting that miR-346-regulated osteogenic differentiation is GSK-3β-dependent.

Our data further demonstrate that miR-346-mediated downregulation of GSK-3β leads to activation of Wnt/β-catenin signaling in hBMSCs. Firstly, miR-346 overexpression significantly increased total and activated β-catenin expression. Secondly, immunofluorescence staining showed increased β-catenin nuclear accumulation in miR-346–overexpressing cells compared to miR-control cells. Thirdly, miR-346 overexpression increased TCF/LEF transcriptional activity, and this effect was blocked by anti-miR-346. In addition, β-catenin knockdown in miR-346-overexpressing cells almost completely blocked the positive effect of miR-346 on osteogenic differentiation. Consistent with these findings, miR-346 overexpression was found to enhance the expression of several β-catenin downstream genes in hBMSCs.

In conclusion, this study identified miR-346 as a positive regulator of human osteogenesis, acting by targeting GSK-3β and activating Wnt/β-catenin signaling in hBMSCs. Our findings suggest that miR-346 may be a useful target in the treatment of pathological conditions of bone loss.
